# Postmortem pancreatic autolysis as a histological marker of early postmortem interval: a forensic autopsy study in a tropical setting

**DOI:** 10.1016/j.fsisyn.2026.100659

**Published:** 2026-01-13

**Authors:** Sunon Sripirom, Vichan Peonim, Wisarn Worasuwannarak

**Affiliations:** Department of Pathology, Faculty of Medicine Ramathibodi Hospital, Mahidol University, Bangkok, Thailand

**Keywords:** Postmortem interval, Time of death estimation, Pancreatic autolysis, Forensic histopathology, Forensic pathology

## Abstract

**Objective:**

To evaluate postmortem pancreatic autolysis as a histological marker of the early postmortem interval (PMI) in routine forensic autopsy, with particular emphasis on regional differences within the pancreas and application in a tropical setting.

**Methods:**

We studied 30 forensic autopsy cases with known PMI <24 h. From each pancreas, tissue from the head, body, and tail was sampled, fixed in formalin, processed routinely, and stained with hematoxylin–eosin. For each region, the proportion of parenchyma exhibiting characteristic autolytic changes was visually estimated as the percentage of autolysis. Spearman's correlation and simple linear regression were used to assess associations between PMI and autolysis.

**Results:**

The PMI ranged from 1 to 17 h (mean 6.4 h). PMI showed a significant positive correlation with autolysis in the head (ρ = 0.508), body (ρ = 0.561), tail (ρ = 0.566) and mean autolysis (ρ = 0.535) (all p ≤ 0.002). Correlations were stronger in traumatic deaths than in non-traumatic deaths. Simple linear regression with PMI as the predictor explained 19.5 % of the variance in mean percent autolysis (R^2^ = 0.195). Autolysis was first appreciable at approximately 2 h postmortem, and >50 % autolysis was not observed below 5 h.

**Conclusions:**

Pancreatic autolysis provides useful information in the early PMI but lacks precision as a stand-alone estimator. It is best used as a practical histological adjunct within a multimodal, evidence-based approach to PMI estimation, especially in warm, humid environments.

## 1 Introduction

Accurate estimation of the postmortem interval (PMI) remains one of the central and most debated tasks in legal medicine because it constrains investigation timelines, helps reconstruct the sequence of events around death, and influences the evaluation of alibis in court [1,2]. Despite persistent methodological advances, PMI assessment in daily forensic practice remains largely based on classical thanatological signs and a limited number of biochemical markers, all of which have significant limitations in terms of precision, environmental dependence, and inter-observer variability [[1], [2], [3]].

Traditional approaches include the assessment of algor, livor, and rigor mortis, as well as the electrolytes of the vitreous humor, particularly potassium, and selected supravital reactions [[1], [2], [3]]. These methods can provide useful information in the early postmortem period, typically within the first 24–72 h after death, but their performance deteriorates when boundary conditions are suboptimal (extreme ambient temperatures, immersion, substantial blood loss or resuscitation, and unusual body habitus) [3,4]. Even under controlled conditions, temperature-based nomograms and muscle or ocular signs rarely yield a narrow time window suitable for high-stakes medico-legal decision-making, and they often require integration with other indicators to achieve acceptable reliability [1,3].

Recent narrative and systematic reviews have highlighted a diversified landscape of novel PMI markers, including biochemical degradation products, RNA and protein decay, immunohistochemistry, postmortem imaging, and microbial community succession [2,[4], [5], [6]]. These techniques show promise in extending useable PMI windows and in refining estimates during specific phases of decomposition. Nevertheless, most of them remain constrained to research settings, require specialized infrastructure or complex modelling, and are sensitive to temperature, humidity, and body storage conditions [2,5,6]. As a result, current expert guidelines emphasize an integrative, evidence-based strategy that combines classical signs with validated adjuncts, rather than relying on any single method [1,5,6].

One of the oldest but still under-standardized adjuncts for PMI estimation is the histological examination of postmortem tissue autolysis. Autolysis, intracellular digestion by endogenous enzymes after cessation of circulation, is organ-specific and progresses at rates determined by intrinsic factors (enzyme content, tissue architecture) and extrinsic factors (temperature, humidity, refrigeration) [7,8]. Experimental work in animal models has demonstrated that highly enzymatic parenchymal organs, such as the pancreas and small intestine, often exhibit early structural changes, whereas more fibrous tissues, like skeletal muscle and myocardium, are comparatively resistant. Histological scoring of autolytic features, including cytoplasmic homogenization, loss of cell borders, nuclear fading, and detachment of epithelia from basement membranes, has therefore been proposed as a potential time marker across various species and organs [[7], [8], [9]].

In humans, systematic histological chronotanatognostic studies remain relatively scarce. Cocariu et al. examined refrigerated cadavers and correlated semi-quantitative autolytic changes in several organs with PMI up to days after death, suggesting reproducible patterns particularly in liver, pancreas, and heart, but with substantial inter-organ variability [10]. Other studies have focused on specific tissues or molecular endpoints, often under experimental or hospital-autopsy conditions, and have underscored that histological autolysis can provide PMI information but is strongly influenced by storage temperature and pre-mortem pathology [11,12]. Despite these insights, histological autolysis has not yet been integrated into routine PMI estimation protocols in forensic casework, in part because the available datasets are small, heterogeneous, and rarely tailored to the early postmortem interval that predominates in medico-legal autopsy practice [2,5,10].

The pancreas is a particularly attractive but underutilized target for such studies. Being a digestive gland rich in proteolytic and lipolytic enzymes, it is widely acknowledged to undergo very rapid autolysis after death [8,13]. Experimental ultrastructural work has shown that the exocrine pancreas exhibits some of the earliest postmortem alterations among major organs in rat models, sometimes within the first hour, with progressive mitochondrial, nuclear, and acinar architectural disruption over the first 24 h [8]. In a multivariate study of human hospital autopsies, Shimizu et al. demonstrated that the severity of pancreatic autolysis was associated not only with PMI but also with clinicopathological conditions such as shock and hyperthermia, further underscoring the organ's sensitivity to both ante- and postmortem influences [14].

More clinically focused work has addressed the practical question of how long useable pancreatic tissue can be obtained after death. In a series of 32 consecutive autopsies from a warm-climate setting, Siriwardana et al. found that the histological architecture of the pancreas (head, body, and tail) was consistently excellent when sampling occurred within 8 h after death, with an increasing proportion of only moderate or poor-quality specimens beyond this threshold [13]. Their findings suggest that pancreatic autolysis not only impairs diagnostic histopathology but may also provide time-graded information within the early postmortem window. However, that study did not derive quantitative autolysis scores or formal PMI prediction models and was based on a relatively limited range of known PMIs, many of them from hospital deaths rather than forensic casework [13,14].

Taken together, existing evidence suggests that pancreatic autolysis is (i) very early and pronounced, (ii) histologically appreciable with routine hematoxylin–eosin (H&E) staining, and (iii) potentially informative for early PMI estimation, especially in warm environments. Yet, there is a conspicuous lack of systematic, quantitative studies of pancreatic autolysis in forensic autopsy populations with well-constrained PMIs. Most prior human studies relied on semi-qualitative grading, mixed a wide range of PMIs, and did not distinguish regional differences between the pancreatic head, body, and tail. They also rarely developed statistical models that could be translated into practical casework tools [10,13,14].

The present study addresses these gaps by analyzing pancreatic autolysis as a supportive histological indicator of the early PMI in a forensic autopsy series. Using systematic sampling of the pancreatic head, body and tail and quantitative estimation of autolytic changes on routine H&E-stained sections, we sought to (i) describe the pattern and regional distribution of pancreatic autolysis within the early postmortem period, (ii) investigate its association with known PMI and relevant case variables, and (iii) explore its potential utility as a practical adjunct for PMI estimation in everyday forensic pathology. We hypothesized that the degree of pancreatic autolysis would increase with PMI in a time-dependent manner and that specific thresholds of histological change could help discriminate short PMI categories in a real-world forensic setting.

## 2 Materials & methods

### 2.1 Study design and setting

This was an observational study of consecutive medicolegal autopsies performed at the Department of Pathology, Faculty of Medicine Ramathibodi Hospital, Mahidol University, Bangkok, Thailand. Data were abstracted from autopsy reports and contemporaneous investigative and medical records.

All cases had a reliably documented time of death and an early postmortem interval (PMI) of less than 24 h. The study evaluated the relationship between histological pancreatic autolysis and PMI in routine forensic casework in a tropical setting.

### 2.2 Case selection and postmortem interval determination

The inclusion criteria comprised consecutive forensic autopsies in which the time of death could be reliably established and the PMI at autopsy was <24 h.

Time of death was determined from:

• Witnessed events (police officers, relatives, other eyewitnesses);
• Medical documentation for hospital deaths (emergency department records, inpatient charts); and
• The contemporaneous assessment of the forensic pathologist based on investigative information and scene findings.

We screened 42 consecutive cases meeting the inclusion criteria. During the interval between death and autopsy (PMI), bodies were maintained at ambient room temperature (∼25 °C) and relative humidity of 50–60 % and were not refrigerated until organ sampling.

Exclusion criteria were pre-defined to minimize confounding by pre-existing pancreatic or systemic pathology. Cases were excluded if there was evidence of:

• Chronic pancreatic disease or other pancreatic pathology diagnosed macroscopically or microscopically at autopsy;
• Systemic infections and sepsis;
• Endocrine system diseases or lysosomal storage disorders;
• The body was refrigerated; or
• Any other condition judged by the forensic pathologist to substantially alter pancreatic morphology or enzymatic activity.

After applying these exclusion criteria, 12 cases were excluded from the study. Totally 30 cases remained for analysis.

### 2.3 Autopsy procedure and pancreatic tissue sampling

All cases underwent standard forensic autopsy according to institutional protocols. From each included case, tissue samples were taken from three anatomically distinct regions of the pancreas: the head, body, and tail.

For each region, two tissue blocks were collected. Samples were immediately immersed in 10 % neutral buffered formalin as a fixative.

After adequate fixation, tissue blocks were trimmed to a standard size of approximately 1 × 1 cm with a depth of 2 mm to ensure comparability across cases and regions.

### 2.4 Histological processing and assessment of autolysis

A total of 180 fixed tissues were processed routinely and embedded in paraffin. Sections were cut at standard histological thickness and stained with hematoxylin and eosin (H&E).

All slides were examined by a forensic pathologist using light microscopy. For each pancreatic region (head, body, and tail), percent autolysis (%) was defined as the proportion of exocrine parenchyma showing one or more of the following autolytic features: (i) loss of acinar architecture; (ii) cytoplasmic homogenization and cell-border indistinctness; (iii) nuclear fading, pyknosis, karyorrhexis, or karyolysis; and/or (iv) detachment or disaggregation of acinar structures. Ducts, vessels, adipose tissue, and stromal elements were excluded from the parenchymal denominator.

Each slide was first scanned at low power to identify representative exocrine parenchyma and to avoid edge or processing artifacts. Percent autolysis was then estimated as a global proportion across the representative exocrine parenchyma of the entire slide at 10 × objective magnification using a standardized visual grid-based area estimation approach (i.e., partitioning the field of view into equal subfields) to approximate the proportion of exocrine parenchyma affected by autolytic change relative to the total assessed exocrine parenchyma, recorded as a percentage (0–100 %). For each region in each case, two tissue blocks were evaluated and averaged to obtain the regional mean. For each case, the overall mean percentage autolysis was calculated by averaging the regional means across all three pancreatic regions.

### 2.5 Variables and data classification

For each case, the following variables were recorded:

• Postmortem interval (PMI): in hours, defined as the time from documented death to pancreatic sampling at autopsy;
• Demographic data: age and sex;
• Manner/circumstances of death: classified as traumatic (accident, homicide, suicide) or non-traumatic (natural disease-related);
• Pancreatic autolysis: percentage autolysis of the head, body, tail, and the mean percentage of the three regions.

The primary outcome was PMI (continuous), and the primary predictors were the regional and overall mean percentages of pancreatic autolysis. Age, sex, and traumatic vs non-traumatic death were considered secondary variables.

### 2.6 Statistical analysis

Continuous variables (PMI and autolysis percentages) were summarized using means and ranges, and categorical variables (sex, traumatic vs non-traumatic deaths) using counts and percentages.

The association between PMI and the degree of autolysis in each pancreatic region (head, body, tail) and the overall autolysis was evaluated using Spearman's rank correlation coefficient, given the non-normal distribution of some variables.

To explore a predictive relationship between autolysis and PMI, simple linear regression analysis was performed with PMI as the independent variable and the mean percentage of autolysis as the dependent variable.

The relationships between categorical or ordinal variables (e.g. sex, age group, traumatic vs non-traumatic deaths) and autolysis were examined using Pearson's chi-square test and Spearman's rank correlation, as appropriate.

A two-sided p-value <0.05 was considered statistically significant. All analyses were performed using SPSS software, version 31.0.1.0 (IBM Corp., Armonk, NY, USA).

## 3 Results

### 3.1 Case characteristics

Thirty forensic autopsy cases met the inclusion criteria for analysis. The age, sex, manner of death, and postmortem interval characteristics are shown in Table 1. Sex, age, and traumatic versus non-traumatic manner of death showed no statistically significant association with the degree of pancreatic autolysis (all p > 0.05).

**Table 1:** Demographic and case characteristics (N = 30).

Variable	Value
Age, years	17–81 (mean 46.7 ± 18.6)
Sex, n (%)	
Male	24 (80.0)
Female	6 (20.0)
Manner of death, n (%)	
Traumatic	19 (63.3)
Non-traumatic	11 (36.7)
Postmortem interval, hours	1–17 (mean 6.4 ± 4.4)
Percent of pancreas autolysis	
Head	0–100 (mean 19.5 ± 26.7)
Body	0–100 (mean 27.1 ± 35.4)
Tail	0–100 (mean 21.7 ± 28.5)
Overall (head–body–tail)	0–100 (mean 22.7 ± 28.9)

### 3.2 Correlation between regional pancreatic autolysis and PMI

The degrees of autolysis in the pancreatic head, body, and tail were highly inter-correlated (Table 2). Spearman's rank correlation coefficients between regions ranged from 0.910 to 0.972 (all p < 0.001), indicating that autolysis progressed relatively synchronously across the gland (Table 2).

**Table 2:** Spearman correlation matrix between regional pancreatic autolysis (N = 30).

Correlation	Head	Body	Tail
Head	–	0.972∗	0.910∗
Body	0.972∗	–	0.912∗
Tail	0.910∗	0.912∗	–

∗p < 0.001 for all listed coefficients.

When correlated with PMI, the severity of pancreatic autolysis showed a statistically significant but modest positive association. Considering the overall mean percentage autolysis across the three regions, the overall correlation with PMI was ρ = 0.535 (p = 0.002). Among individual regions, autolysis in the pancreatic tail demonstrated the highest correlation with PMI (Table 3).

**Table 3:** Spearman's rank correlation between regional pancreatic autolysis and PMI.

Part of the pancreas	Total deaths (p) (N = 30)	Traumatic deaths (p) (N = 19)	Non-traumatic deaths (p) (N = 11)
Head	0.508 (0.004)	0.626 (0.004)	0.378 (0.252)
Body	0.561 (0.001)	0.675 (0.002)	0.343 (0.301)
Tail	0.566 (0.001)	0.627 (0.004)	0.446 (0.169)

Overall (head–body–tail)	0.535 (0.002)	0.648 (0.003)	0.372 (0.260)

When cases were stratified by manner of death, the correlations between autolysis and PMI were generally stronger in traumatic deaths than in non-traumatic deaths. In traumatic cases (n = 19), the correlation between autolysis and PMI was ρ = 0.648 (p = 0.003), with similarly elevated coefficients for the head, body, and tail. In non-traumatic cases (n = 11), the corresponding correlations were lower and did not reach statistical significance; for overall mean autolysis, ρ = 0.372 (p = 0.260). This pattern suggests that acute deaths may provide a clearer temporal signal of pancreatic autolysis than deaths associated with chronic disease processes (Table 3).

### 3.3 Regression analysis and empirical thresholds

**Fig. 1:**
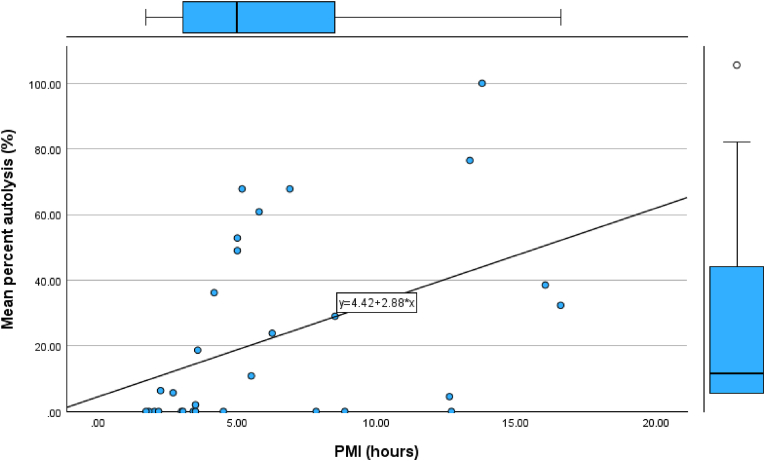
Scatterplot showing the relationship between PMI (hours) and mean percentage autolysis of the pancreas (%) in 30 cases, with fitted linear regression line and 95 % confidence band.

Fig. 1 illustrates the association between postmortem interval (PMI, hours) and mean percent pancreatic autolysis (%) (averaged across the head, body, and tail). A positive trend was observed, with higher autolysis values occurring at longer PMIs; however, substantial dispersion was also evident, indicating heterogeneity in autolysis at similar PMIs.

On simple linear regression with mean percent autolysis (%) as the dependent variable and PMI (hours) as the predictor (n = 30), the model fit was statistically significant (R = 0.442; R^2^ = 0.195; adjusted R^2^ = 0.167; F(1,28) = 6.80; p = 0.014), with a standard error of estimate of 26.38. The fitted regression equation was:

Mean percent autolysis (%) = 4.418 + 2.878 × PMI (hours)

PMI was a significant predictor (B = 2.878, SE = 1.104; β = 0.442; t = 2.607; p = 0.014), corresponding to an average increase of approximately 2.9 percentage points in mean autolysis per additional hour postmortem. Despite statistical significance, the modest explained variance and scatter around the fitted line suggest that histological autolysis, as measured here, should be interpreted as an adjunct indicator of early PMI rather than a standalone estimator.

From an empirical standpoint, pancreatic autolysis was first observed at approximately 2 h after death in this series. Moreover, pancreatic samples with more than 50 % autolysis were associated with a PMI of at least 5 h (p = 0.01 by Pearson's chi-square test); no cases with >50 % autolysis occurred below this threshold. This suggests that autolysis of the pancreas may be useful for defining a minimum PMI (e.g. “unlikely to be < 5 h”) rather than for providing a narrow, exact time estimate.

## 4 Discussion

This study examined pancreatic autolysis as a potential histological marker of the early postmortem interval (PMI) in a real-world forensic autopsy series from a tropical setting. Within a PMI range of 1–17 h, we found a statistically significant, positive association between the PMI and degree of pancreatic autolysis. Simple linear regression using PMI as the predictor explained approximately 20 % of the variance in autolysis, and pronounced autolysis (>50 %) was only observed at PMIs of 5 h or greater. These findings suggest that pancreatic autolysis carries useful timing information for early postmortem periods but has limited precision as a stand-alone estimator.

### 4.1 Relationship to current PMI estimation strategies

Recent overviews of PMI research emphasize that no single method provides uniformly accurate estimates across all conditions, and that traditional signs (algor, rigor, livor), biochemical markers, and emerging molecular or microbial approaches each cover specific time windows and have context-dependent limitations [5,[15], [16], [17]]. Advanced techniques, such as omics-based profiling, volatile organic compound (VOC) analysis, and microbial succession, can achieve high accuracy in experimental models but often require specialized equipment, controlled environments, and complex modeling, which may not be available in routine casework [5,15,18,19].

Against this background, histological assessment of postmortem autolysis remains an attractive, low-cost adjunct that uses infrastructure already present in most medico-legal centers. Our results support the concept that organ-specific autolytic change, in this case, of the pancreas, can contribute quantitative information about early PMI, particularly in tropical climates where classical temperature-based nomograms are difficult to apply reliably.

### 4.2 Comparison with previous autolysis and pancreatic studies

In the present study, pancreatic autolysis was operationalized as a continuous, area-based histological metric, expressed as the percentage of exocrine parenchyma demonstrating predefined autolytic features (loss of acinar architecture, cytoplasmic homogenization with indistinct borders, nuclear fading or dissolution, and/or acinar detachment/disaggregation). This proportional approach was selected because it provides an intuitive and analytically tractable measure that can be applied consistently across regions and cases, allowing correlation analysis and regression modeling while preserving information that would be lost with coarse ordinal grading. The workflow, initial low-power scanning to select representative parenchyma and avoid edge or processing artifacts, followed by estimation at 10 × objective magnification using a standardized visual, grid-like partitioning of the field of view, was intended to improve internal consistency and comparability across samples in routine forensic practice.

Several authors have investigated autolytic change as a time marker in human and animal models. Cocariu et al. quantified autolysis in refrigerated cadavers up to 22 days postmortem and found that pancreas, liver, and myocardium showed progressive, organ-specific changes, with the pancreas among the earliest and most severely affected organs [10]. Their work confirmed that the pancreas is highly sensitive to postmortem time but dealt predominantly with longer PMIs in cold storage. By contrast, our series focuses on PMIs <24 h, most of which involve an initial interval at ambient temperatures of ∼25 °C, thereby addressing the short-term and warm-climate range that is common in forensic practice but underrepresented in the literature.

Shimizu et al. examined 92 human pancreases and used multivariate analysis to show that PMI was the single most important determinant of the extent of autolysis, but that other clinicopathological factors (mode of death, malignancy, prior surgery) significantly modified the autolytic pattern [14]. Their model achieved an R^2^ of 0.71 when incorporating 12 covariates, underscoring both the potential and the complexity of interpreting pancreatic autolysis. Our more modest R^2^ (≈0.20) using only the percentage of autolysis as a predictor is consistent with Shimizu's conclusion that PMI is necessary but not sufficient to explain the degree of pancreatic autolysis, especially when other variables are not modelled explicitly.

Siriwardana et al. addressed a related but distinct question, namely the optimal time window for obtaining diagnostically useful pancreatic histology after death in a warm-climate hospital setting [13]. They reported excellent histological quality in all specimens obtained within 8 h and an increased proportion of moderate/poor slides thereafter, suggesting that autolysis significantly degrades diagnostic quality beyond this point. Our findings complement theirs by quantifying the degree of autolysis across pancreatic regions and formally correlating these measurements with PMI. The observation that >50 % autolysis was not seen below 5 h aligns with their empirical 8-h quality threshold, and together these data indicate that the pancreas is both a vulnerable organ for diagnostic histology and a promising substrate for early PMI estimation in warm climates.

Our observation that correlations between pancreatic autolysis and PMI were stronger in traumatic than in non-traumatic deaths is consistent with broader forensic literature showing that the agonal period and premorbid status substantially modulate postmortem tissue changes [20,21]. In sudden traumatic deaths, rapid circulatory arrest with minimal pre-existing organ compromise tends to produce a relatively “clean” temporal signal, in which autolysis progresses predominantly as a function of postmortem time and environmental conditions [21]. By contrast, non-traumatic deaths often arise from chronic cardiac, respiratory, or systemic disease, sepsis, or malignancy, which can alter pancreatic perfusion, enzyme content, fibrosis, and cellular resilience before death, thereby blurring the relationship between PMI and autolytic severity [8,14,20]. Shimizu et al. demonstrated that clinicopathological factors such as malignancy and shock significantly influenced pancreatic autolysis independent of PMI, supporting the concept that disease-related “background noise” can attenuate the time signal in hospital and natural deaths [14]. Taken together, these findings suggest that pancreatic autolysis may be most informative for early PMI estimation in sudden, relatively uncomplicated deaths, and that interpretation in non-traumatic or chronic-disease contexts should be made cautiously and, where possible, adjusted for relevant clinical and pathological factors.

### 4.3 Forensic applicability

From a practical perspective, the relatively low R^2^ of our regression model and the wide scatter of data points around the fitted line mean that pancreatic autolysis cannot provide precise, case-level PMI estimates on its own. Instead, the value of this marker lies in its ability to support or constrain broader PMI intervals derived from other methods. Our data suggest two potential applications:

1. **Minimum PMI thresholds.** The absence of >50 % autolysis in below 5 h PMI in our series indicates that marked autolysis may help exclude very short PMIs (e.g. “unlikely to be < 5 h”), which can be useful when evaluating competing investigative scenarios.
2. **Contextual corroboration in early PMI.** When traditional signs and investigative information suggest an early PMI in a warm, humid environment, the presence of only mild pancreatic autolysis may support such an assessment, whereas unexpectedly advanced autolysis could prompt re-evaluation of the assumed timeline or consideration of modifying factors (e.g. pre-existing disease, hyperthermia, delayed refrigeration).

Current reviews stress that robust PMI estimation should integrate multiple lines of evidence rather than rely on a single marker [5,[15], [16], [17]]. In this sense, pancreatic autolysis is best conceived as one component within a multimodal approach that may also include body cooling curves, biochemical markers, imaging, microbial or omics signatures, and scene/investigative data, tailored to the case context and available resources.

### 4.4 Limitations and future directions

This study has several limitations that must be acknowledged. First, the sample size was relatively small (30 cases) and drawn from a single institution, which limits the generalizability of our findings. Second, the percentage of autolysis was estimated using a visual, area-based approach by a single observer; inter- and intra-observer variability were not formally assessed, and we did not employ digital image analysis or computer-assisted overlays that could improve reproducibility and facilitate cross-center harmonization. Third, we did not construct multivariable models incorporating potential confounders, such as detailed agonal course, comorbidities, or medication history, which Shimizu et al. have shown can substantially affect pancreatic autolysis [14]. Finally, our analysis focused on linear relationships and did not evaluate more complex modeling approaches (e.g., nonlinear regression, machine learning) that are increasingly used in other PMI research domains, such as VOC profiling, microbiome analysis, and omics-based markers.

Future studies should therefore aim to:

• Enroll larger, multi-center forensic cohorts across a broader range of environmental conditions;
• Document ambient and storage conditions in detail, including humidity and refrigeration parameters;
• Use standardized histological scoring systems with inter-observer testing and/or digital image analysis; and
• Integrate pancreatic autolysis with other PMI indicators in multivariable or machine-learning frameworks to explore whether overall models can yield more precise and generalizable estimates.

Such work would clarify whether pancreatic autolysis can move from a promising adjunct to a validated component in evidence-based PMI protocols.

## 5 Conclusions

In this forensic autopsy series from a tropical setting, we found that the degree of pancreatic autolysis shows a statistically significant positive association with the early postmortem interval. Autolysis was first appreciable at approximately 2 h after death, and marked autolysis (>50 %) was not observed until 5 h postmortem, suggesting that pancreatic histology can provide time-graded information within the first 24 h postmortem. However, the modest proportion of PMI variance explained by autolysis alone and the wide scatter of individual data points indicate that pancreatic autolysis lacks the precision to serve as a stand-alone PMI estimator.

Rather than a single “time of death calculator”, pancreatic autolysis should be regarded as a practical adjunct within a multimodal, evidence-based approach to PMI estimation. In cases from warm, humid environments, where classical temperature-based methods are difficult to apply and advanced molecular tools may be unavailable, quantitative assessment of pancreatic autolysis on routine H&E sections may help exclude very short PMIs and support broader time intervals derived from other findings. Larger, multi-center studies with rigorous control of environmental factors, standardized scoring, and integration with additional markers are warranted to clarify the definitive role of pancreatic autolysis in contemporary forensic practice.

## CRediT authorship contribution statement

**Sunon Sripirom:** Writing – original draft, Visualization, Project administration, Methodology, Investigation, Formal analysis, Data curation, Conceptualization. **Vichan Peonim:** Writing – review & editing, Supervision, Methodology, Investigation, Formal analysis, Conceptualization. **Wisarn Worasuwannarak:** Writing – review & editing, Validation, Supervision, Methodology, Formal analysis.

## Ethics approval

The research protocol was approved by the Ethical Clearance Committee on Human Rights Related to Research Involving Human Subjects, Faculty of Medicine Ramathibodi Hospital, Mahidol University (MURA2017/122), which waived the requirement for informed consent.

## Funding

The authors did not receive support from any organization for the submitted work.

## Declaration of competing interest

The authors declare that they have no known competing financial interests or personal relationships that could have appeared to influence the work reported in this paper.
